# Interaction of Heavy Metal Ions with Carbon and Iron Based Particles

**DOI:** 10.3390/ma7032242

**Published:** 2014-03-18

**Authors:** Dana Fialova, Monika Kremplova, Lukas Melichar, Pavel Kopel, David Hynek, Vojtech Adam, Rene Kizek

**Affiliations:** 1 Department of Chemistry and Biochemistry, Mendel University in Brno, Zemedelska 1, CZ-613 00 Brno, Czech Republic; E-Mails: dana.dospivova@seznam.cz (D.F.); mkremplova@volny.cz (M.K.); meldalm@gmail.com (L.M.); paulko@centrum.cz (P.K.) d.hynek@email.cz (D.H.); vojtech.adam@mendelu.cz (V.A.); 2 Central European Institute of Technology, Brno University of Technology, Technicka 3058/10, CZ-616 00 Brno, Czech Republic

**Keywords:** electrochemical detection, multi-wall nanotubes, graphene, expanded carbon, heavy metal ions, paramagnetic particle, voltammetry

## Abstract

Due to the rapid development of industry and associated production of toxic waste, especially heavy metals, there is a great interest in creating and upgrading new sorption materials to remove these pollutants from the environment. This study aims to determine the effectiveness of different carbon forms (graphene, expanded carbon, multi-wall nanotubes) and paramagnetic particles (Fe_2_O_3_) for adsorption of cadmium(II), lead(II), and copper(II) on its surface, with different interaction time from 1 min to 24 h. The main attention is paid to the detection of these metals using differential pulse voltammetry. Based on the obtained results, graphene and Fe_2_O_3_ are found to be good candidates for removal of heavy metals from the environment.

## 1. Introduction

Metal ions are still a threat, they pollute environment and have great bioaccumulation potential [1–3]. Heavy metals can enter the human body through the digestive or respiratory tract and their effects may be very serious. Heavy metals can cause a disruption of function of the kidneys, bones, central nervous system, and hematopoietic system and have adverse biochemical, histological, neuropsychological and reproductive effects [4].

Designing new technological methods for effective removal of heavy metals from the environment is the leading point in a considerable number of research institutions [5–10]. In recent years, nanotechnologies have recorded much development that has the potential to be used also in this case [11]. These highly advanced and revolutionary technologies are focused on the study and application of materials based on individual particles having a size in the order of nano and/or micrometers [12,13].

For isolation of heavy metals, it is possible to use different materials with high sorption properties that are able to adsorb metal ions onto their surface or into their structure. Different modifications of carbon, such as graphene, nanotubes, or fullerenes are important members of this group [14]. In addition various carbon modifications, paramagnetic particles (PMPs) with comparable properties can also be used [13]. Paramagnetic particles are particles sized from units to tens of microns. Due to their physical and chemical properties, they are widely used, especially in biomedical and biotechnological research fields [15–17]. Modification of paramagnetic particles significantly affects their behavior and binding specificity of the target analyte. Paramagnetic properties of particles are mediated by modification of surface using maghemite (γ -Fe_2_O_3_), which covers the surface of particles and corresponds to the insulation quality. By modification of surface coverage, the amount of bound analyte as well as the behavior of paramagnetic particles can easily be increased or decreased. The combination of two selective bioanalytical processes, namely the specific binding of analytes onto the surface of particles by molecular recognition and a specific isolation using magnetic fields from complex mixed samples, makes paramagnetic particles an effective tool in many ways [18,19]. The ability to respond to an external magnetic field is also attractive because of the possibility of easy immobilization of analyte [20].

Fast, sensitive, and simple analytical determination of metal ions in the environment is very important. The determinations can be performed by a variety of instrumental methods [12,13,21–25]. Usually, the presence of trace amounts of heavy metals in the environmental samples is detected by spectrophotometric techniques such as atomic absorption spectrometry (AAS) and/or mass spectrometry with inductively coupled plasma (ICP–MS) [6,9,26]. However, these techniques require complex laboratory equipments with limited availability and highly qualified staff. On the other hand, the electrochemical methods have the best metal detection limits, sufficient selectivity for metal ions, low costs, high sensitivity and mobility. Another advantage is the use of various electrochemical methods as pulse voltammetry and/or chronopotentiometry [27–32].

This study was focused on the isolation of cadmium, lead, and copper ions from solution using adsorbents, such as graphene, multi-wall carbon nanotubes (MWCNTs), expanded carbon, and iron paramagnetic particles with subsequent metal detection by electrochemical methods.

## 2. Results and Discussion

Carbon-based substances and iron magnetic particles were used as adsorbents for the removal of heavy metals. The surface of these materials has excellent sorption properties, which were used for adsorption of cadmium, lead, and copper.

### 2.1. Determination of Cadmium(II), Lead(II) and Copper(II) Ions

**Figure 1.**
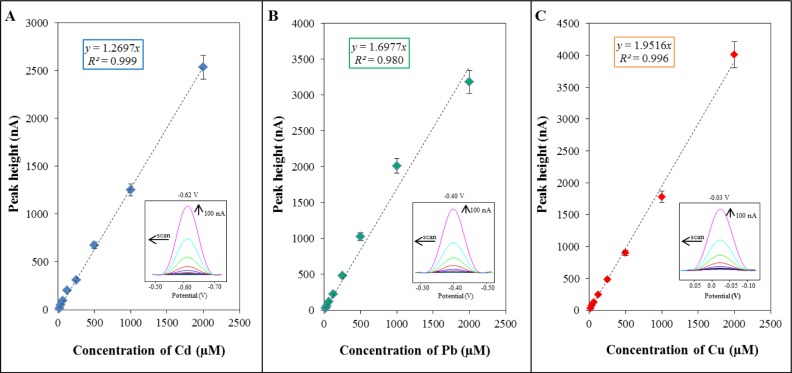
Calibration curves of individual metals determined by differential pulse voltammetry: (**A**) cadmium; (**B**) lead and (**C**) copper. 0.2 M acetate buffer (pH = 5) was used as an electrolyte. The parameters were chosen as it follows: initial potential −1.3 V, end potential 0.2 V, deposition potential −1.15 V, accumulation time 240 s, pulse amplitude 25 mV, pulse time 0.04 s, voltage step 5.035 mV, voltage step time 0.3 s, and sweep rate 0.0168 V/s. The characteristic peaks for cadmium, lead and copper were measured at potentials of −0.62, −0.40 and −0.03 V, respectively.

For verification and evaluation of the sorption properties, differential pulse voltammetry was used. This method was employed for the detection of metal ions as a highly sensitive technique [33,34]. For determination of individual metal ions, standard solutions of Cd(NO_3_)_2_·4H_2_O, Pb(NO_3_)_2_ and Cu(NO_3_)_2_·3H_2_O in the concentration range of metal ions from 15 to 2000 μM were utilized. The concentration dependence obtained for cadmium was linear within the range from 15 to 2000 μM of cadmium ions (*y* = 1.2697*x; R*^2^ = 0.999, Figure 1A). A characteristic peak of cadmium was detected at a potential of −0.62 V. The concentration dependence obtained for lead was linear within the range from 15 to 2000 μ M of lead ion with equation as it follows *y* = 1.6977*x; R*^2^ = 0.980 (Figure 1B). The characteristic peak of lead was measured at a potential of −0.40 V. The concentration dependence obtained for copper was linear within the range from 15 to 2000 μM of copper ion with equation as it follows *y* = 1.9516*x; R*^2^ = 0.996 (Figure 1C). A characteristic peak of copper was detected at a potential of −0.03 V. It follows from the obtained results that the method is the most sensitive for cadmium ions.

### 2.2. The Effectiveness of the Used Adsorbents for Cd, Pb, and Cu Sorption

In this study, four types of adsorbents for the sorption of cadmium, lead and copper ions were investigated. Three of them were carbon-based (reduced graphene oxide, expanded carbon, and MWCNTs) and the last one was represented by iron magnetic particles (Fe_2_O_3_). The sorption was carried out at pH 7 as the most common condition in environment. The other pH values were not tested. All the three metals were expected to be dominantly present in their divalent ionic forms [35]. The interaction time of metals with adsorbents was studied from 1 min to 24 h (1, 5, 10, 15, 30 min, 1, 3, 6, 12 and 24 h).

**Figure 2.**
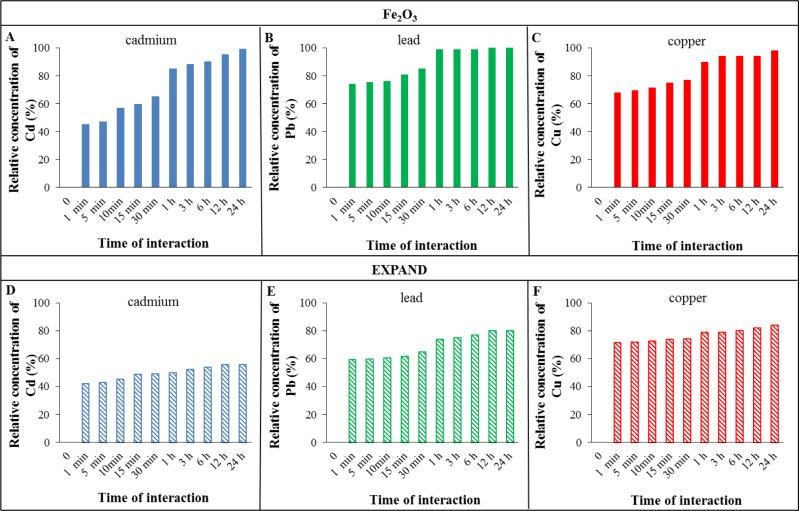
The amount of bounded cadmium (**A**,**D**); lead (**B**,**E**) and copper (**C**,**F**), on various adsorbents: (**A**,**B**,**C**) for Fe_2_O_3_; (**D**,**E**,**F**) for expanded carbon. All values were related to the applied concentration of metal (100 μM for cadmium, lead and copper). Zero on the x-axis is control.

**Figure 3.**
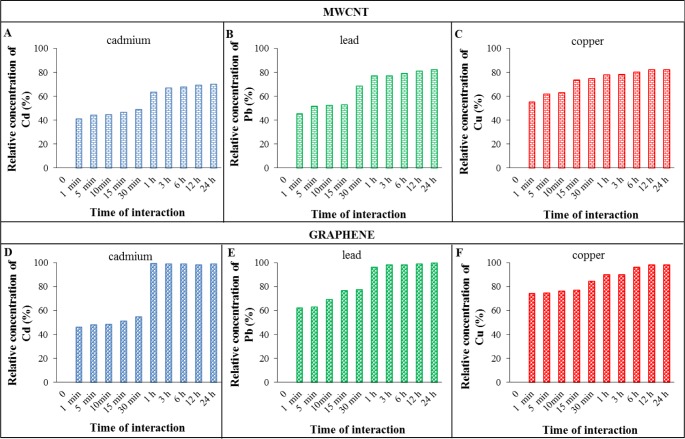
The amount of bounded cadmium (**A**,**D**); lead (**B**,**E**) and copper (**C**,**F**), on various adsorbents: (**A**,**B**,**C**) for multiwall nanotubes; (**D**,**E**,**F**) for reduced graphene oxide. All values were related to the applied concentration of metal (100 μM for cadmium, lead and copper). Zero on the x-axis is control.

Changes in cadmium, lead and copper adsorption efficiencies of the studied adsorbents are shown in Figures 2 and 3. The concentrations of the metal ions after adsorption at selected times were measured in the filtrate. The columns height represents the absolute efficiency, which is the difference between the applied concentration (100 μ M for all metals) and the concentration detected after metal adsorption at a certain time. These values were used to obtain the values of absolute efficiency. The amounts of metals detected in the filtrate were subtracted from the applied concentrations of metals (100 μ M).

Graphical trends show increasing adsorption of metal ions with the increase in time of interaction with adsorbents. Comparing the metal adsorption efficiencies at two different interaction times (30 min and 24 h) suggest Fe_2_O_3_ to be the best adsorbent for all metals at 30 min and both Fe_2_O_3_ and graphene for 24 h interaction time. Specifically, the best adsorbents for individual metals were as follows: for cadmium and lead, it was Fe_2_O_3_ for 30 min interaction time and both Fe_2_O_3_ and reduced graphene oxide at 24 h. For copper, it was reduced graphene oxide for 30 min interaction time and Fe_2_O_3_, MWCNTs and reduced graphene oxide for 24 h. The results show that the reduced graphene oxide and Fe_2_O_3_ have the highest capacities to adsorb metal ions on their surfaces.

After 30 min of interaction, the adsorption efficiencies of reduced graphene oxide and Fe_2_O_3_ for cadmium were 55% and 65% respectively. At 24 h interaction time, the adsorption efficiencies for these adsorbents increased to ~100% while the values for expanded carbon (56%) and MWCNTs (70%) were considerably lower. A similar trend was observed for lead. The adsorption efficiencies of reduced graphene oxide and Fe_2_O_3_ at 30 min were 77% and 85% respectively which increased to 100% after 24 h. The trend for copper adsorption on various adsorbents was different than cadmium and lead. At 30 min interaction time, the efficiency values were not significantly different for Fe_2_O_3_ (77%), expanded carbon (74%) and MWCNT (74%) whereas the efficiency was comparatively higher for reduced graphene oxide (84%). After 24 h, the copper adsorption efficiency increased to 98% for Fe_2_O_3_, MWCNT and reduced graphene oxide and 84% for expanded carbon. These results also show that lead is adsorbed better than cadmium on all the four adsorbents. This may be attributed to the higher electronegativity value of lead. Electronegativity plays an important role in controlling the adsorption of metal ions. The more electronegative metals tend to form stronger covalent bonds with oxygen atoms located on nanoparticles surfaces. According to the Pauling’s scale of electronegativity, these metals ions should be adsorbed preferably in the order of Pb, Cu, Cd [36]. The data indicates reduced graphene oxide and Fe_2_O_3_ to be the better adsorbents than expanded carbon and MWCNT. This is attributable to the presence of functional groups (−COOH, −OH, −O) with free electron pairs on their surface which may lead to the formation of strong covalent or coordination bonds [37].

### 2.3. Statistical Evaluation of Experimental Data

**Figure 4.**
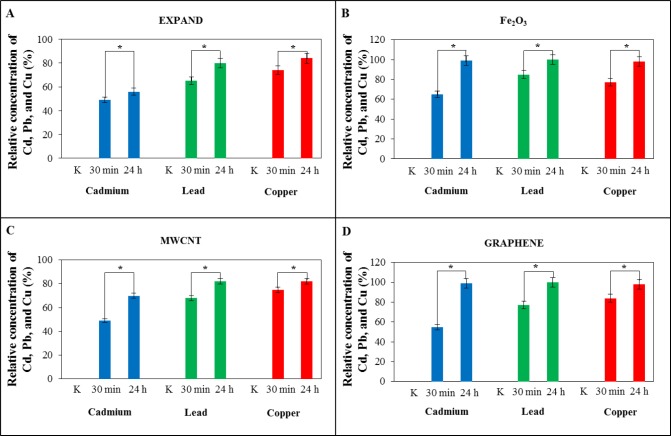
The comparison of time interaction of heavy metal with different adsorbents (**A**) expanded carbon; (**B**) MPs Fe_2_O_3_; (**C**) multi-walled carbon nanotubes and (**D**) graphene (reduced graphene oxide). All values were related to the applied concentration of heavy metals (100 μM for cadmium, lead, and copper). K on the x-axis is control. * Statistically significant at the significance level of *p* < 0.05.

Statistical evaluation of experimental data should be an integral part of the results. It is important to determine if the results are statistically significant or not. In Figure 4, the adsorption efficiency of used adsorbents for cadmium, lead, and copper is compared in each graph as Figure 4A for expanded carbon, Figure 4B for Fe_2_O_3_, Figure 4C for MWCNT and Figure 4D for reduced grapheme oxide. For graphical representation, two interaction times, 30 min and 24 h, were selected. All values were related to the applied concentration of the metal ions (100 μ M). The value of adsorption efficiency for 24 h was chosen as 100% and the value for 30 min was correlated to 100% value.

Statistical analysis was applied on these two values for all metals and adsorbents. In data analysis, it was found that the results are statistically significant at the significance level of *p* < 0.05. It was established that between the compared results for 30 min and 24 h of interaction, there is a difference of more than 5% at our chosen significant level, and different effectiveness of adsorption which is also apparent from the graph.

The statistical analysis confirmed differences of the values in the efficiency of sorption (time of interaction 30 min and 24 h) as significant. For cadmium, the least significant difference in the effectiveness was evaluated for expanded carbon and the most significant for reduced graphene oxide. On the other hand, for lead, the least significant difference was evaluated for Fe_2_O_3_ particles and the most significant for reduced graphene oxide. For the copper, the least significant difference in the effectiveness is generally for the MWCNTs and the most significant for Fe_2_O_3_ particles.

### 2.4. Determination of Concentration Capacity at Adsorption of Reduced Graphene Oxide and Fe_2_O_3_

**Figure 5.**
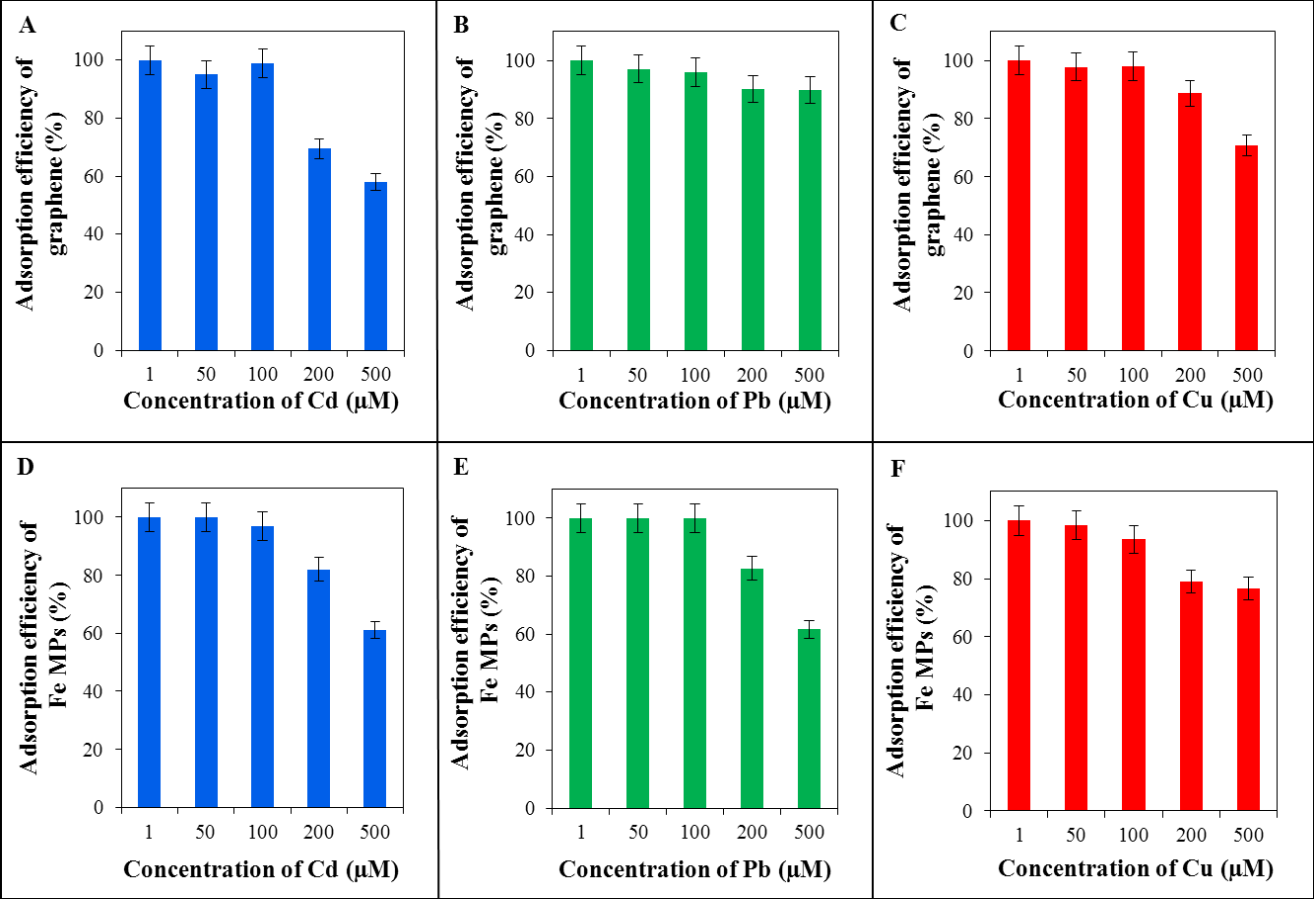
Determination of the concentration of adsorbent capacity of graphene (reduced graphene oxide): concentration capacity (**A**) for cadmium; (**B**) for lead and (**C**) for copper and of Fe_2_O_3_ MPs: concentration capacity (**D**) for cadmium; (**E**) for lead and (**F**) for copper. Efficiency of adsorption for each metal is plotted on the y-axis %. Applied concentrations of metals (Cd^2+^, Pb^2+^, and Cu^2+^) were 1, 50, 100, 200 and 500 μ M.

The concentration capacity of selected adsorbents was determined using differential pulse voltammetry. Based on the measured and evaluated data, the value of concentration, which is the limit for both adsorbents (reduced graphene oxide in Figures 5A–C; and Fe_2_O_3_ particles in Figures 5–F), was determined. The limit value of concentration is 100 μ M. The efficiency of adsorption was calculated according to the formula: Absorption efficiency = 100% − (*C_D_*/*C_V_*) × 100%. *C_D_* is the detected concentration of metal in the filtrate. *C_V_* is the bounded concentration of metal. With application of increasing concentration of metal ions, the efficacy of adsorption decreased. The reason for the reduced efficiency of adsorption with the increasing concentration of the metal is probably the formation of a monolayer on the surface of adsorbent.

The adsorption mechanism of metal ions could take place through different reaction mechanisms, including surface adsorption, ion exchange and/or by creating covalent bonds [35]. The surfaces of reduced graphene oxide and maghemite are somewhat similar. Both types of adsorbents contain oxygen functional groups. Reduced graphene oxide contains an oxygen, hydroxyl and carboxyl group on its surface, according to the applied method of reduction [37]. In contrast, the maghemite contains hydroxyl groups. These groups consist of surface hydroxyl groups that usually arise from water adsorption or from structural OH. The surfaces of metal oxides in aqueous solution are generally attached with hydroxyl groups that can change in form at different pH values. These groups contain a double pair of electrons together with a dissociable hydrogen atom that can generate suitable conditions for them to react with both acids and bases. The charge on the iron oxide surface dominates the adsorption or desorption of protons and it is generated by the dissociation (ionization) of the surface hydroxyl groups depending on the pH of the solution [38]. In our case, the pH value was set at 7 and therefore the presence of metals in its divalent ionic forms is expected. The differentiation among various metal sorption effects is not aim of this study and therefore no experiments leading for such conclusion were estimated. The surface adsorption of divalent metal ions is almost connected with ion exchange reaction between metal ions in solution and proton bonded on the particle surface. The electrostatic interaction is a weak interaction compared with chemical adsorption. The pH values may affect the effect of adsorption just due to the change of surface charge [36,39–43]. The structural loading of metal ions is, in contrast, linked with the filling of vacancies in structural lattice [35,44,45].

## 3. Experimental Section

### 3.1. Chemicals

FeCl_3_·6H_2_O, FeCl_2_·6H_2_O, and other chemicals were purchased from Sigma Aldrich (Sigma-Aldrich, St. Louis, MA, USA) unless noted otherwise. Stock solutions were prepared with ACS water. pH value and conductivity were measured using an inoLab Level 3 (Wissenschaftlich-Technische Werkstatten GmbH, Weilheim, Germany). Deionized water underwent demineralization by reverse osmosis using an Aqua Osmotic 02 (Aqua Osmotic, Tisnov, Czech Republic) and was subsequently purified using a Millipore RG (MiliQ water, 18 M Ω, Millipore Corp., Billerica, MA, USA). Deionized water was used for rinsing, washing, and buffer preparation.

### 3.2. Preparation of Graphene

The first step of reduced graphene oxide preparation was according to the standard method of Hummers [46]. The graphite (2 g) was added to 46 mL of concentrated sulfuric acid and mixed by stirring and cooled with ice, followed by an addition of 1 g NaNO_3_ and 6 g of KMnO_4_. The mixture was left 24 h at laboratory temperature, in order to thicken it. All the black graphene oxide was stirred in 300 mL of ACS water. To this suspension, 4 mL of 35% hydrazine and 32 mL of 25% NH_3_ were added. After that, the mixture was heated on a water bath for 60 min, cooled, and washed with 1200 mL of water, and sucked on a frit. Finally, methanol was added. Again, suction and drying in a vacuum desiccators at 40 °C was carried out [37,47].

### 3.3. Preparation of Expanded Carbon

Natural graphite was mixed with sulfuric acid in a 1:1 weight ratio. Formed mixture was oxidized with hydrogen peroxide (50%) in a stainless reactor under vigorous reaction. After the reaction, the graphite material was put in a muffle/vacuum furnace and allowed to expand at a temperature of 850 °C for 30 s. Natural graphite increased many times in volume during expansion. The conductivity of this material depends on the size of flakes created during the expansion [48].

### 3.4. Preparation of Fe_2_O_3_ MPs

5.4 g FeCl_3_·6H_2_O and 2 g FeCl_2_·6H_2_O were dissolved in 40 mL of water. Both the chlorides were transferred to 250 mL of 1.5 M NaOH (15 g NaOH). Resulting black precipitate was sonicated for 10 min (SONOREX digital 10P, Bandelin, Berlin, Germany) and separated magnetically. After washing with water and drying at 40 °C, the yield was 0.47 g. Annealing was carried out in a muffle furnace at 400 °C for about 1 h [49].

### 3.5. Preparation of Samples to Isolate Metal Ions

**Figure 6.**
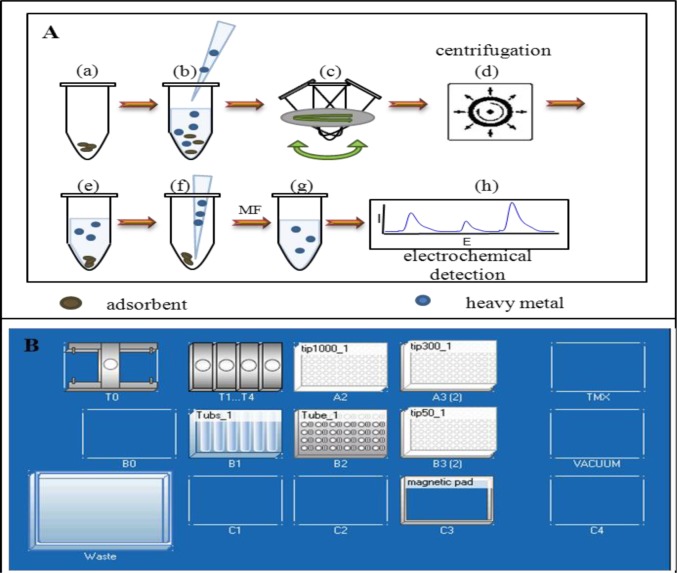
(**A**) Preparation of samples: (a) weighed adsorbent; (b) pipetting solution of heavy metal; (c) shaking the sample; (d,e) centrifugation of the sample; (f) pipetting of supernatant; (g) filtration with a MF (membrane filter); (h) electrochemical detection of heavy metal using differential pulse voltammetry. (**B**) Automatic preparation of samples on EpMotion 5075 (Eppendorf, Germany)—scheme of instrumentation workplace. T0 carrier (capacity ≤ 1200g), T1…T4 dosing machines: TS 1000, TS 300, TS 300/8, TS 50, tip1000_1 epTIPS Motion 1000 μ L, tip300_1 epTIPS Motion 300 μ L, tip50_1 epTIPS Motion 50 μ L, Tubs_1 Holder Eppendorf with 7 × 30 mL reservoirs (max volume: 30 mL, working volume: 25 mL, limit of detection: 3000 μL), Tube_1 Rack Eppendorf, Magnetic pad—magnet for MPs, TMX—thermomix with temperature control, VACUUM—vacuum.

To 10 mg of adsorbent (expanded carbon, Fe_2_O_3_, reduced graphene oxide, multi-wall nanotubes) 1 mL of solution of heavy metal (100 μM) was pipetted. Interaction of metal and the adsorbent was carried out in five different time intervals (1, 5, 10, 15, and 30 min). Subsequently, we also tested longer period of interaction (1, 3, 6, 12, and 24 h) under shaking (BIOSAN, Multi RS-60) at room temperature. After the interaction, the sample was centrifuged (BIOSAN, FVL 2400N, Combi-Spin) for 10 min. The supernatant was carefully removed using a syringe with a needle and filtered through a membrane filter (0.45 μm). Metal was detected in the sample by differential pulse voltammetry. Manual preparation of the sample is schematically shown in Figure 6A and automatic preparation in Figure 6B.

#### Automatic Procedure of Samples Preparation

For automated sample preparation, an automatic pipetting station epMotion 5075 (Eppendorf) was used. This equipment is controlled with suitable program for precise application of solutions to adsorbents of heavy metals and subsequent compliance the interaction time with stirring. Working area for automated preparation of sample (Figure 6B) is organized as it follows: T0 is the carrier for dispensing machines to pipette prepared solutions of heavy metals to the pre-weighed adsorbents. Sucking the solutions takes place in the position B1-Tubs_1 to preselected positions on a rackTube_1. Replacement of tips for pipetting arm is divided by the volume in the positions A2, A3 and B3. After using, the tips were automatically inserted to the waste box. TMX position is for mixing the solution. In this position, it is possible to select and control the desired temperature. A magnet is located in the position C3 and it serves for working with magnetic particles. The particles are tightened by the magnet and subsequently sucked off the sample. The automatic pipetting station EpMotion accelerated the preparation of the sample.

### 3.6. Electrochemical Determination of Cadmium, Lead, and Copper Ions

Determination of cadmium, lead, and copper by differential pulse voltammetry was performed using a 797 VA Stand (Metrohm) and a standard cell with three electrodes. The three electrode system consisted of a hanging mercury drop electrode (HMDE) with a drop area of 0.4 mm^2^ as the working electrode, the Ag/AgCl/3M KCl reference electrode and a platinum wire as the auxiliary electrode. GPES 4.9 software was employed for data processing. The analyzed samples were deoxygenated prior to measurements by purging with argon (99.999%). Deoxygenation in the electrochemical detection of metals was done due to the removal of oxygen from the measured sample solution. The presence of oxygen could cause disturbance in obtained voltammograms.

Acetate buffer (0.2 M CH_3_COONa + CH_3_COOH, pH 5) was used as a supporting electrolyte. The supporting electrolyte was replaced after each analysis. The parameters of the measurement were as it follows: purging time 120 s, initial potential ¬1.3 V, end potential 0.2 V, deposition potential ¬1.15 V, accumulation time 240 s, pulse amplitude 25 mV, pulse time 0.04 s, voltage step 5.035 mV, voltage step time 0.3 s, sweep rate 0.0168 V/s, volume of injected sample: 15 μL, volume of measurement cell 2 mL (15 μ L of sample; 1985 μ L acetate buffer). The characteristic peak for cadmium was measured at a potential of −0.62 V, for lead and copper at potentials of −0.40 V and −0.03 V respectively [50].

### 3.7. Testing Concentration Capacity of Reduced Graphene Oxide and Fe_2_O_3_ Particles

In order to test the best adsorption properties, reduced graphene oxide and Fe_2_O_3_ were chosen. For determination of the concentration capacity, 10 mg of adsorbent was applied. 1 mL of solution of cadmium, lead, and copper in various concentrations was added to the adsorbent. Concentration of metals was as it follows: 1, 50, 100, 200, and 500 μM. The time of interaction was 1 h. To prepare these samples, the same procedure like preparing samples for time interaction was used.

### 3.8. Descriptive Statistics

Software STATISTICA (data analysis software system), version 10.0 (Tulsa, OK, USA) was used for data processing. General regression model was used to analyze differences between the combinations of compounds. To reveal differences, Turkey’s post hoc test within homogenous groups was employed. Unless noted otherwise, *p* < 0.05 was considered significant.

## 4. Conclusions

Fast and efficient removal of heavy metals from the environment is an important. In our study, we examined both the synthesis and applications of adsorbents. Four types of absorbents (reduced graphene oxide, expanded carbon, carbon nanotubes, and magnetic particles Fe_2_O_3_) were tested for adsorption of cadmium, lead, and copper ions. It has been found that reduced graphene oxide and Fe_2_O_3_ MPs have higher adsorption efficiency for all tested metals than the other two carbon materials.
